# Changes in primary metabolism and associated gene expression during host-pathogen interaction in clubroot resistance of *Brassica napus*

**DOI:** 10.1371/journal.pone.0310126

**Published:** 2024-09-09

**Authors:** Aleya Ferdausi, Swati Megha, Nat N. V. Kav, Habibur Rahman

**Affiliations:** Department of Agricultural, Food and Nutritional Science, University of Alberta, Edmonton, AB, Canada; Nuclear Science and Technology Research Institute, ISLAMIC REPUBLIC OF IRAN

## Abstract

The role of primary metabolism during *Brassica napus*-*Plasmodiophora brassicae* interaction leading to clubroot resistance has not yet been investigated thoroughly. In this study, we investigated some of the primary metabolites and their derivatives as well as expression of the genes involved in their biosynthesis to decipher this host-pathogen interaction. For this, two sets (clubroot resistant and susceptible) of canola lines were inoculated with *P*. *brassicae* pathotype 3A to investigate the endogenous levels of primary metabolites at 7-, 14-, and 21-days after inoculation (DAI). The associated pathways were curated, and expression of the selected genes was analyzed using qRT-PCR. Our results suggested the possible involvement of polyamines (spermidine and spermine) in clubroot susceptibility. Some of the amino acids were highly abundant at 7- or 14-DAI in both resistant and susceptible lines; however, glutamine and the amino acid derivative phenylethylamine showed higher endogenous levels in the resistant lines at later stages of infection. Organic acids such as malic, fumaric, succinic, lactic and citric acids were abundant in the susceptible lines. Conversely, the abundance of salicylic acid (SA) and the expression of benzoate/salicylate carboxyl methyltransferase (*BSMT*) were higher in the resistant lines at the secondary stage of infection. A reduced disease severity index and gall size were observed when exogenous SA (1.0 mM) was applied to susceptible *B*. *napus*; this further supported the role of SA in clubroot resistance. In addition, a higher accumulation of fatty acids and significant upregulation of the pathway genes, glycerol-3-phosphate dehydrogenase (*GPD*) and amino alcohol phosphotransferase (*AAPT*) were observed in the resistant lines at 14- and 21-DAI. In contrast, some of the fatty acid derivatives such as phosphatidylcholines represented a lower level in the resistant lines. In conclusion, our findings provided additional insights into the possible involvement of primary metabolites and their derivatives in clubroot resistance.

## Introduction

Plants can recognize pathogen attacks and deploy defense responses through reprogramming their primary and secondary metabolic pathways. Primary metabolic pathways provide energy and carbon skeletons that are required for the host to activate defense responses due to pathogen infection [1]. The metabolites produced during this defense response act as molecular signals inducing changes to plant primary metabolism, leading to the production of secondary metabolites related to plant resistance or susceptibility [2]. The primary metabolites such as amino acids, organic acids and fatty acids are biosynthesized through complex pathways that involve several enzymes [1]. Unlike transcriptomics and proteomics, metabolomics serves as a global ‘omics’ platform leading to the detection and quantification of metabolites. These metabolites are well-reported for identifying and signaling the morphological, physiological and biochemical responses that take place during pathogen infection [2].

Clubroot disease of *Brassica* crops, caused by *Plasmodiophora brassicae* Woronin, is a major concern worldwide. In oilseed *B*. *napus*, this disease can result in a yield loss of about 30% and can also decrease seed oil content by about 2–6% [for review, see 3,4]. The primary zoospores of this pathogen invade host cells through root hairs establishing primary infection without exhibiting any visible disease symptoms. Secondary infection is caused by the secondary zoospores invading the root cortex, and this results in the formation of visible clubs or galls [5]. During disease progression, the pathogen relies on the host’s primary metabolism for nutrients and energy [6]. The galls constitute a durable metabolic sink of amino acids, sugars, organic acids and lipids limiting their transportation to the other parts of the plant resulting in an altered host metabolism and stunted growth [6]. Pathogen infection alters the expression of a number of genes involved in the biosynthesis or degradation of carbohydrates, amino acids and lipids [for review, see 7].

Polyamines are amine-containing, low molecular weight molecules that are formed by aliphatic hydrocarbons replaced with multiple amino groups [8]. They are biosynthesized directly from amino acids such as arginine, proline and ornithine [8]. Polyamines are the major constituents of plant cell walls and are responsible for cell proliferation and differentiation, hence play a potential role during pathogenic gall formation [8]. They are also well-reported for their involvement in biotic and abiotic stress responses and play an important role as crucial barriers to pathogen invasion [for review, see 9]. The most common polyamines found in plants i.e., spermidine, spermine and their diamine precursor putrescine are considered to be major constituents of plant cells, and they contribute to the development and regulation of resistance [for review, see 8].

Among the primary metabolic pathways, amino acid metabolism in response to pathogen infection has been well-studied in many plant species [for review, see 7] including in *B*. *napus* due to infection by *P*. *brassicae* [10,11]. For instance, Wagner et al. (2012, 2019) [10,11] reported a positive correlation of clubroot susceptibility with the club-root induced accumulation of amino acids such as asparagine and alanine. The changes in the endogenous levels of other primary metabolites [7,11] such as amines, amino acids, organic acids, lipids and fatty acids in host tissues due to pathogen infection are intriguing; however, they have not been investigated so far in the *B*. *napus-P*. *brassicae* pathosystem.

The present study was, therefore, undertaken to investigate the role of primary metabolites such as polyamines, amino acids and their derivatives, organic acids and fatty acids in mediating clubroot resistance in canola. Additionally, based on the changes in metabolites we also investigated the changes in expression of the genes associated with the metabolic pathways that are involved in the biosynthesis of these metabolites. The results from this study extend our knowledge about the role of specific metabolites in *P*. *brassicae* pathosystem and may provide a better understanding of the energy-producing primary metabolism fueling the resistance response or susceptibility in canola.

## Materials and methods

### Plant materials

A set of clubroot resistant and a set of clubroot susceptible spring *B*. *napus* canola doubled haploid (DH) lines were used in this study–each set included 12 lines. The DH lines were produced from F_1_ plants of a cross involving a clubroot resistant canola line, carrying resistance of the rutabaga (*B*. *napus* var. *napobrassica*) cv. Polycross, and a susceptible canola line. The details of the development of these lines including their resistance to *P*. *brassicae* pathotype 3A can be found in Wang et al. (2022) [12].

### Inoculation with *P*. *brassicae*

Plants of the resistant and susceptible DH lines were grown in a greenhouse at 22/15°C (day/night) temperature, 16 h photoperiod, and light intensity of 130 μmol m^-2^ s^-1^ at plant level in a completely randomized design and were inoculated with single spore isolate of *P*. *brassicae* pathotype 3A, as classified on the Canadian Clubroot Differential set [3]. This isolate was obtained from Dr. Stephen Strelkov, Department of Agricultural, Food and Nutritional Science, University of Alberta in the form of gall. Resting spore suspension of the isolate was prepared following the protocol described by Hasan et al. (2012) [13]. Briefly, frozen galls carrying *P*. *brassicae* pathotype 3A were homogenized in distilled water and filtered through eight layers of cheesecloth (American Fiber & Finishing Inc., Albemarle, NC, United States) to obtain the spore suspension. The concentration of the spores in the suspension was adjusted to 1 × 10^6^ spores/ml with a haemocytometer (VWR, Mississauga, ON) [13]. Inoculation was done 10 days after seeding by pipetting 1 mL inoculum at the base of the seedlings [13]. To ensure successful infection, the seedlings were inoculated on the following day using the same procedure.

### Sample collection

Root samples of the inoculated resistant and susceptible plants were collected at 7-, 14- and 21-days after inoculation (DAI); samples of uninoculated (control) resistant and susceptible plants were also collected at the same time points. These time points were selected based on our observation that the primary infection in canola plants carrying rutabaga-resistance occurs at 6- to 14-DAI and the secondary infection occurs at 14- to 18-DAI [14]. For each time point, three plants of each of the 12 resistant and 12 susceptible lines were used, and the samples of the 36 plants (3 × 12) for each time point and group were bulked separately. The experiment was carried out in three replications; thus, the total number of bulks was 36 (2 groups × 3 time points × 2 treatments × 3 replications), and the total number of plants grown for this experiment was 36 bulks × 2 groups × 36 plants = 2,592. The samples were stored at -80°C and were used for both metabolomics and quantitative real-time PCR (qRT-PCR) analysis. The metabolites were quantified by The Metabolomics Innovation Centre (TMIC) at the University of Alberta, Canada.

### LC-MS/MS analysis of primary metabolites

#### Root tissue extraction

The list of chemicals, preparation of stock solutions, internal standard mixtures (ISTDS), and root tissue extraction procedure can be found in Zheng et al. (2021) [15]. Briefly, homogenized freeze-dried samples (25 mg) were placed in 1.5 ml Eppendorf tubes, and 1 ml of pre-cooled hexane and methanol (3:1, v/v) mixture was added to the tubes and vortexed at 1000 rpm (Fisherbrand™ vortexer, Ottawa, CA, USA) for 10 min at 4°C. After adding 500 μl of 25% aqueous methanol, the tubes were sonicated on an ice bath for 10 min, vortexed again for 1 min and centrifuged at 10,000 RCF (relative centrifugal force or × g) for 10 min at 4°C. After centrifugation, two different layers of root extract i.e., aqueous and hexane layers were obtained and aliquoted into two separate Eppendorf tubes.

#### Extract analysis

Three different protocols involving two different pre-column derivatization reactions (PITC; phenylisothiocyanate and 3-NPH; 3-nitrophenylhydrazines) were required for the targeted analysis of amino acids, lipids and organic acids, and this included the PITC derivatization for amino acids, DFI (direct flow injection) buffer dilution and mixing for lipids, and 3-NPH derivatization for organic acids [15]. Briefly, for the PITC analysis, 10 μl of the sample solution, including 50% aqueous methanol as a single blank, calibration standards and quality control solutions [15], and aqueous root extracts along with 20 μl of ISTD mixture solutions were pipetted individually on each spot of a 96-well Multiscreen Solvinert Filter Plate. The filter plate was then dried under a gentle nitrogen steam for 30 min, and 50 μl of 5% PITC derivatization solution was added to each well. The plate was placed at room temperature for 20 min followed by further drying under a gentle nitrogen steam for 1 hr. Subsequently, 300 μl of extraction solution (ES) prepared with 5 mM ammonium acetate in methanol was pipetted in each well. The filter plate was covered and agitated on a rotary shaker for 30 min at 450 rpm and then centrifuged at 50 RCF for 5 min to collect the sample extract to a Nunc® 96 DeepWell™ plate. 150 μl sample extract was mixed with 150 μl distilled water and the samples were then analyzed by LC-MS/MS to quantify the amino acids.

For the quantification of lipids, 5 μl sample solution, which included 50% aqueous methanol as a single blank, quality control solutions and root extracts from the hexane layer were mixed with 10 μl of ISTD mixture and pipetted to a Nunc® 96 DeepWell™ plate. Afterwards, 150 μl of extraction solution (ES) was added to each well followed by the addition of 600 μl of DFI buffer solution containing 60 μl of formic acid, 10 ml of water and 290 ml of methanol. The samples were then analysed by direct flow injection-tandem mass spectrometry (DFI-MS/MS).

In the 3-NPH derivatization method, 10 μl of ISTD mixture and 10 μl sample solution, as prepared for the PITC assay, were added to each well of a Nunc® 96 DeepWell™ plate. Afterwards, 30 μl of 75% aqueous methanol was added to each well followed by the addition of 25 μl of three solutions viz., 250 mM of 3-NPH in 50% aqueous methanol, 150 mM EDC (1-ethyl-3-(3-dimethylaminopropyl) carbodiimide) in methanol, and 7.5% pyridine solution dissolved in 75% aqueous methanol. The plate was then shaken at 450 rpm for 2 h at room temperature and 350 μl water and 25 μl BHT (butylated hydroxytoluene) solution (2 mg/ml in methanol) were added to each well. The samples were then analysed by LC-MS/MS to quantify the organic acids.

### LC/DFI-MS/MS analysis

LC/DFI-MS/MS analyses for the quantification of amino acids, organic acids and lipids were performed using an Agilent 1260 series UHPLC system (Palo Alto, CA, USA) coupled with an AB Sciex QTRAP® 4000 mass spectrometer (Concord, ON, USA). An Agilent reversed-phase Zorbax Eclipse XDB C18 column (3.0 mm × 100 mm, 3.5 μm particle size, 80 Å pore size) attached to a Phenomenex (Torrance, CA, USA) SecurityGuard C18 pre-column (4.0 mm × 3.0 mm) and a Red PEEK tubing connected to the LC and MS system was used. The Analyst® 1.5.3 software was used for analysis of the samples while Analyst ® 1.6.3 and MultiQuant 3.0.3 software were used for data analyses [15]. The instrument parameters and column settings were followed as reported by Zheng et al. (2021) [15].

### Data acquisition, calculation and analysis

The calibration regression of the LC/MS/MS analysis assays was evaluated using seven standard mixture solutions to generate a seven-point calibration curve for the quantification of amino acids and organic acids. The peak area for each metabolite was plotted against its corresponding isotope labeled ISTD peak area to generate a calibration curve, and this was used to determine the coefficient of determination (*R*^*2*^) for each metabolite. A representative single-point calibration curve was used for the metabolites lacking individual chemical standards considering linear regression through zero. The details of the method validation, calibration regression and calculations are described by Zheng et al. (2021) [15].

Metabolite concentrations (μM/g DW; dry weight) were obtained from TMIC and data were analyzed using MS Excel and MetaboAnalyst 5.0 (www.metaboanalyst.ca). Normalization of data for ANOVA was performed using mean and Pareto scaling; *p-*value threshold was set to 0.05 and Fisher’s LSD was used for post-hoc analysis. The sample groupings were performed using multivariate PCA analysis by MetaboAnalyst 5.0. The metabolites were also analyzed using MetPA [16] to postulate their corresponding pathways and the enzymes were curated using the Kyoto Encyclopedia of Genes and Gnomes database (KEGG; www.genome.jp/kegg/).

### Gene expression analyses

#### RNA extraction and cDNA synthesis

Total RNA of the root bulks stored at -80°C was extracted using TRIzol reagent (Invitrogen, USA) and DNase treatment was done using DNase I, RNase-free kit following manufacturer’s protocol (ThermoFisher Scientific, USA). RNA concentration and quality of each sample were analyzed using a Nanodrop 2000 spectrophotometer (ThermoFisher Scientific, USA). RevertAid First Strand cDNA Synthesis Kit (ThermoFisher Scientific, USA) was used for cDNA synthesis following manufacturer’s instructions and the cDNA samples were stored at -80°C.

#### Primer design

The polyamines, amino acids, organic acids and fatty acids were mapped to their corresponding pathways to identify the pathway enzymes/genes. The *Arabidopsis* homologues for the pathway genes were retrieved from The Arabidopsis Information Resource (TAIR; www.arabidopsis.org/), and the homologues were mapped to the *B*. *napus* genome using the *B*. *napus* Genome Browser of Genoscope (www.genoscope.cns.fr/brassicanapus/) to identify their physical positions and to design gene-specific primers. The primers for 19 genes were designed using the software program Primer3Plus (www.bioinformatics.nl/cgi-bin/primer3plus/primer3plus.cgi). The list of the primers is presented in S1 Table.

#### Quantitative Reverse-Transcription PCR (qRT-PCR)

qRT-PCR of the samples was carried out with two technical replicates comprising six replicates for each sample (3 biological × 2 technical) on a StepOne Plus real-time PCR system (Life Technologies, Burlington, Canada) using FASTSYBR Green mix (ThermoFisher Scientific, USA). The housekeeping gene Ubiquitin-Conjugating Enzyme 10 (*UBC10*) from *B*. *napus* was used as endogenous control. The relative expression level of each gene was calculated using the 2^− ΔΔCt^ method [17]. Data were analyzed using MS Excel and statistical analysis was performed using ANOVA with post-hoc Tukey HSD Test (astatsa.com/OneWay_Anova_with_TukeyHSD/).

### Treatment of plants with salicylic acid (SA)

SA solutions of 0.5 mM, 1.0 mM, 2.5 mM and 5.0 mM were prepared using SA (suitable for plant cell culture, Sigma Aldrich) dissolved in ethanol/distilled water (v/v 1:1). The resistant and susceptible lines, which were used for metabolomics study, were grown in a greenhouse and inoculated with *P*. *brassicae* pathotype 3A following the protocol as described above. After 10–12 DAI, 1 ml of SA was directly pipetted to the base of the plants for 4 consecutive days and the plants were grown for another 35 days. Thereafter, plants were uprooted, washed and scored on a 0–3 scale [18] based on their gall formation, where 0 = no galls, 1 = a few small galls on the lateral roots, 2 = moderate galls, and 3 = severe galls. By using the resistance scores, disease severity index (DSI) was calculated using the following formula [19]:

$$DSI(\%)=\frac{\sum (n_{0}\times 0+n_{1}\times 1+n_{2}\times 2+n_{3}\times 3)}{N\times 3}\times 100$$

where *n*_*0*_, *n*_*1*_, *n*_*2*_ and *n*_*3*_ are the number of plants included in the disease severity classes 0, 1, 2 and 3, respectively, and *N* is the total number of plants evaluated.

## Results

### Differential regulation of polyamines in canola roots due to *P*. *brassicae* inoculation

**Fig 1:**
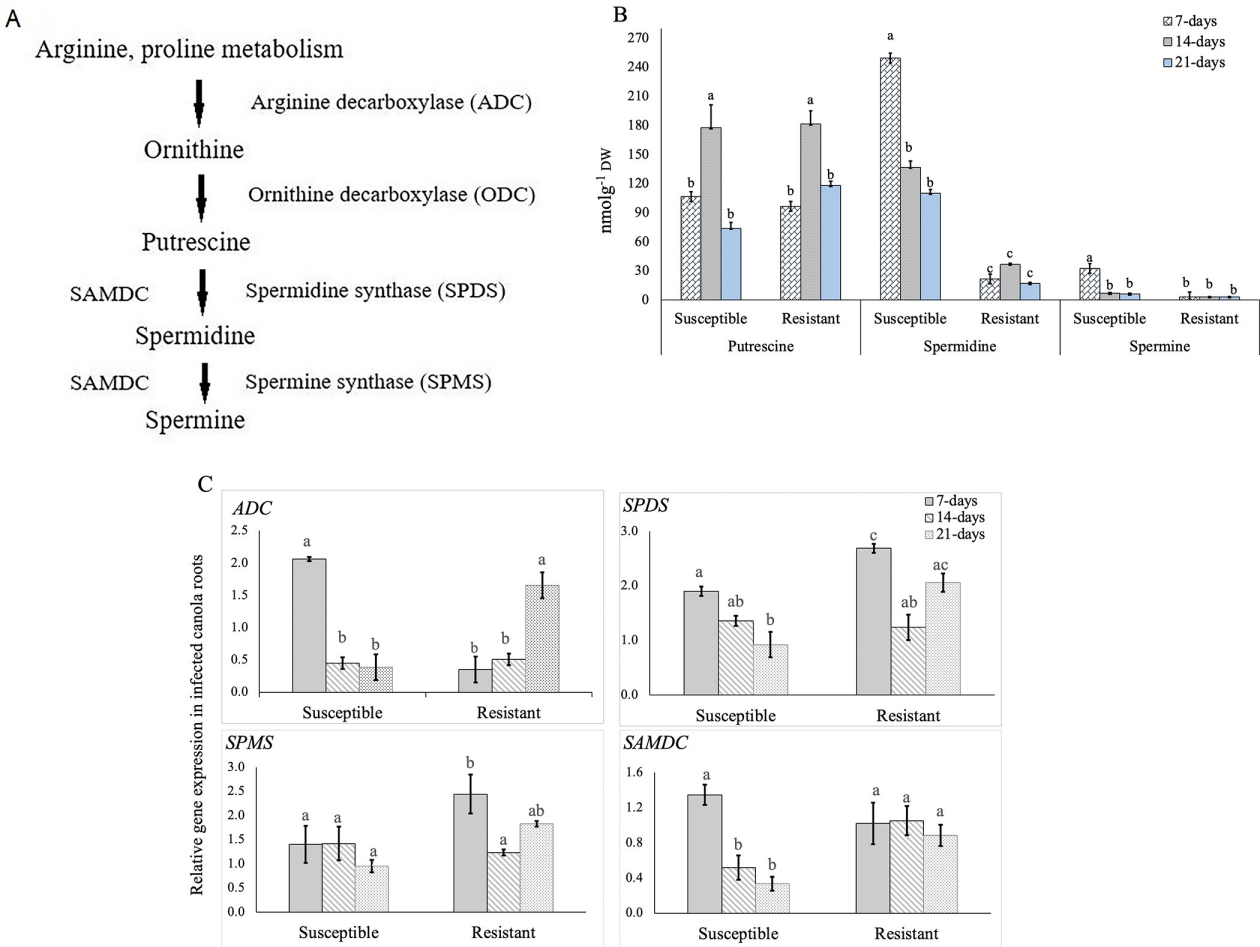
A: Pathway showing the enzymes ADC (arginine decarboxylase), ODC (ornithine decarboxylase), SPDS, SPMS and SAMDC involved in the biosynthesis of the polyamines. B: Endogenous levels of different polyamines in root samples of the susceptible and resistant *B*. *napus* canola lines at 7-, 14- and 21-days after inoculation (DAI) with *P*. *brassicae* pathotype 3A. C: Relative expression of the polyamine biosynthetic genes in root samples of the susceptible and resistant *B*. *napus* canola lines at 7-, 14- and 21-DAI with *P*. *brassicae*. Gene expression levels were normalized to the corresponding gene expression (1.0) in the uninoculated controls.

The polyamines detected in canola root samples were putrescine, spermidine and spermine biosynthesized from arginine and proline (Fig 1A). No significant differences in the accumulation of these polyamines could be detected between the non-infected (control) resistant and susceptible lines. However, the mean concentration of these three polyamines was slightly higher in the susceptible lines than in the resistant lines (S2 Table). In case of the inoculated samples, the content of putrescine was similar in both resistant and susceptible lines (Fig 1B). However, a significantly (*p* < 0.05) greater content of spermidine, the downstream metabolite in the biosynthetic pathway of polyamines (Fig 1A), was found in roots of the susceptible lines at all three time points as compared to the resistant lines (Fig 1B). A similar pattern was also observed for spermine, even though the content of this metabolite was significantly lower than the endogenous levels of putrescine and spermidine (Fig 1B).

The postulated polyamine biosynthesis pathway shows that the genes *SPDS* (spermidine synthase) and *SAMDC* (S-adenosyl-methionine decarboxylase) are involved in the biosynthesis of spermidine and spermine from their precursor putrescine (Fig 1A). qRT-PCR analysis showed a decreased expression of these two genes as the infection progressed in the susceptible lines (Fig 1C), while their expression remained constantly high at all three time points in the resistant lines. The reasons for the stable and high expression of *SPDS*, *SPMS* (spermine synthase) and *SAMDC* in the resistant lines but the decreased contents of spermidine and spermine in the susceptible lines require further investigation.

### Changes in amino acid profiles as a result of *P*. *brassicae* infection

A total of 26 amino acids and their derivatives were detected in the infected and non-infected canola roots; among them, 19 were selected based on their concentration and One-way ANOVA post-hoc (MetaboAnalyst 5.0) analysis (*p*-value < 0.01) for further study (S3 Table).

### Principal Component (PCA) analysis and clustering (Heatmap)

**Fig 2:**
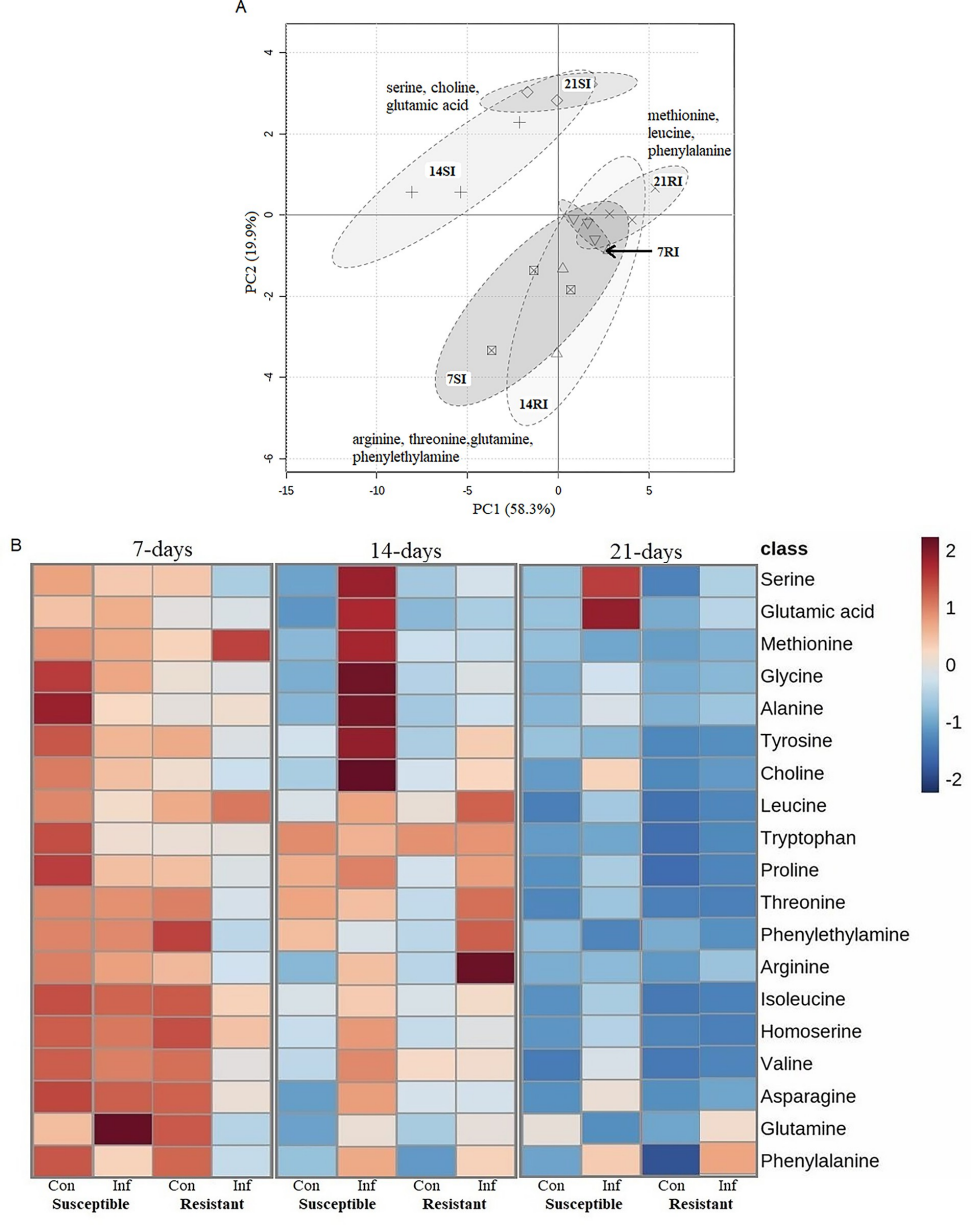
A: Principal component analysis, PCA (PC1 *versus* PC2), score scatter plot of the amino acids and their derivatives of the *B*. *napus* root samples collected from clubroot susceptible (7-SI, 14-SI and 21-SI) and resistant lines (7-RI, 14-RI and 21-RI) at 7-, 14- and 21-days after inoculation (DAI) with *P*. *brassicae* pathotype 3A. B: Heatmap representing the amino acids and their derivatives detected in roots of the control (Con) and *P*. *brassicae* pathotype 3A-inoculated (Inf) resistant and susceptible *B*. *napus* canola lines, and their relative abundance (high to low color gradient box on the top right) at three time points: 7-, 14- and 21- DAI.

PCA analysis revealed a total of 95.8% variations in the dataset of the inoculated root samples of the resistant and susceptible lines, where PC1 and PC2 accounted for 58.3% and 19.9% of the total variation, respectively (Fig 2A). Based on this, the samples were grouped into six clusters where the inoculated samples (SI) of the susceptible lines at 14- and 21-DAI (14-SI and 21-SI) fell into two overlapping clusters on positive and negative loadings of the two PCs. The metabolites assigned in this separation were serine, choline and glutamic acid. All other four clusters were also overlapped, where the inoculated samples of the susceptible lines at 7-DAI (7-SI) and inoculated resistant (RI) lines at 14-DAI (14-RI) were closely associated along both positive and negative loadings of the PC1. The important amino acids for the separation of these clusters were arginine, threonine, glutamine and phenylethylamine (Fig 2A). The inoculated root samples of the resistant lines at 7- and 21-DAI (7RI and 21RI) were closely associated and overlapped with the 7-SI and 14-RI samples; the assigned amino acids for these clusters were methionine, leucine and phenylalanine (Fig 2A).

ANOVA analysis (Heatmap) showed that the endogenous levels of 17 amino acids, and phenylethylamine and choline were significantly different among the control and inoculated root samples collected over three time points (Fig 2B). In the case of the inoculated samples, a significantly greater accumulation of serine, glutamic acid, methionine, glycine, alanine, tyrosine and choline was observed in the susceptible samples at 14-DAI where the concentration of serine and glutamic acid remained high at 21-DAI. On the other hand, relatively higher amounts of leucine, tryptophan, threonine, phenylethylamine and arginine were detected in the resistant samples at 14-DAI. However, the endogenous levels of these metabolites were decreased at 21-DAI (Fig 2B).

**Fig 3:**
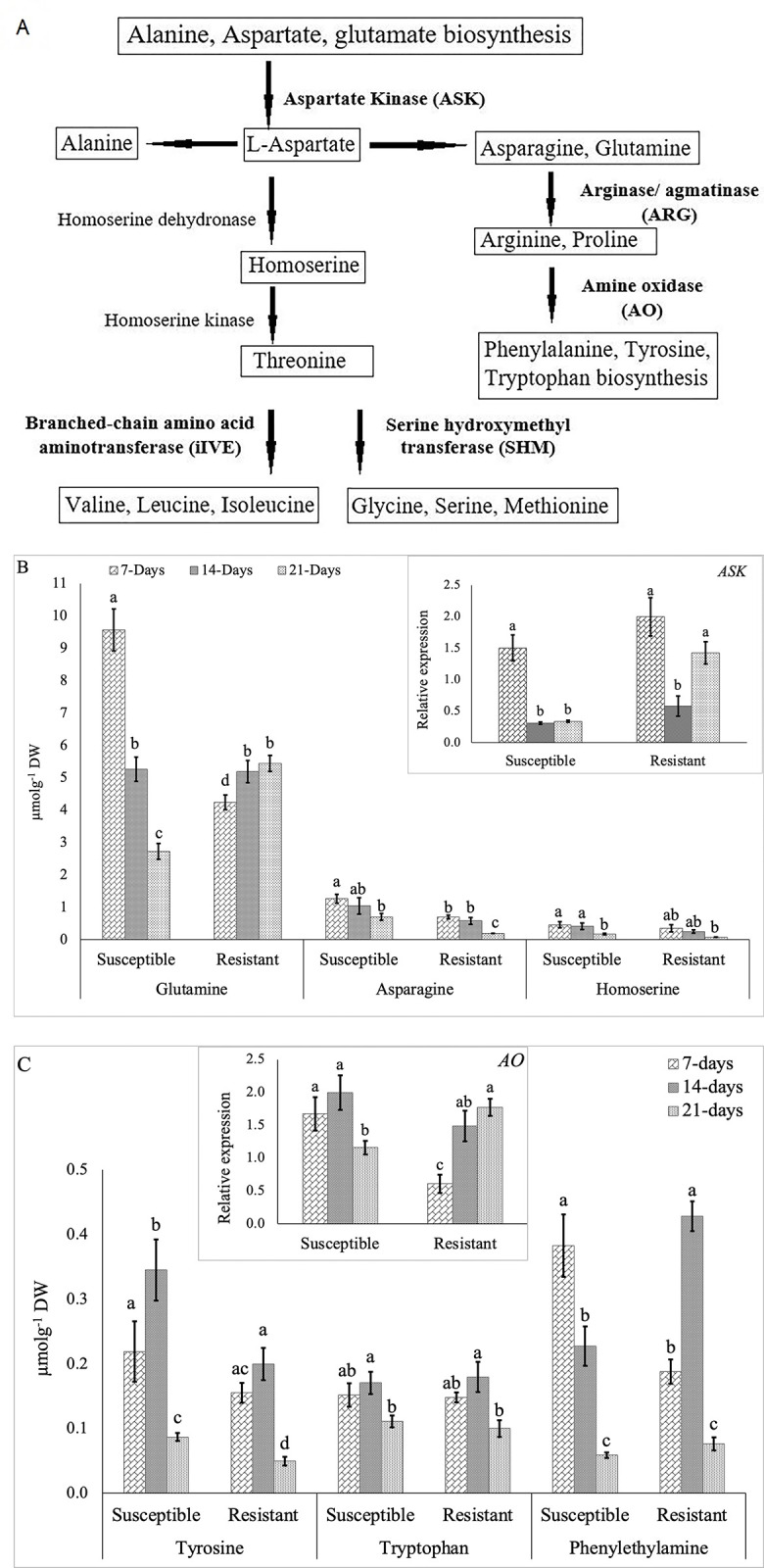
Amino acid and other metabolites profiling of the infected root samples of the susceptible and resistant *Brassica napus* canola lines at 7-, 14- and 21-days after inoculation (DAI) with *Plasmodiophora brassicae* pathotype 3A. A: Biosynthetic pathway along with the important enzymes involved (bold). B: Accumulation of *ASK* leading amino acids and relative expression of *ASK* (right top corner). C: Accumulation of *AO-*leading metabolites and relative expression of *AO* (top). The gene expression levels were normalized to the corresponding gene expression (1.0) in the control sample i.e. without inoculation.

Pathway analysis using MetPA represented six metabolic pathways as the most enriched pathways (S4 Table) for the aforementioned 17 amino acids, phenylethylamine and choline. Their postulated biosynthetic pathways and the associated enzymes as potentially having an important role in mediating clubroot resistance are represented in Fig 3A. The endogenous levels of asparagine and homoserine were significantly (*p*-value < 0.05) decreased in both resistant and susceptible inoculated roots with the progression of infection. A similar trend, as mentioned above, was also observed for glutamine in the susceptible lines; however, its endogenous level exhibited an opposite trend in the resistant lines (Fig 3B). Expression analysis of the genes from the postulated metabolic pathways for the amino acids showed a decreased expression of the early pathway gene *ASK* (aspartate kinase) in the susceptible lines over the three time points. This observation was also correlated with the decreasing abundance of glutamine, asparagine and homoserine at these time points (Fig 3B). However, the expression pattern of this gene in the resistant lines varied as the infection progressed, where it decreased at 14-DAI but increased at 21-DAI to a level statistically similar to the level that was observed at 7-DAI (Fig 3B).

A significant reduction in the endogenous levels of phenylethylamine, which is derived from amino acids phenylalanine and tyrosine, was observed in the susceptible lines over the time points after inoculation, where the content of this compound at 21-DAI was about 6-fold lower than the content at 7-DAI (Fig 3C). The gene *AO* (amine oxidase) involved in the biosynthesis of phenylalanine also showed the lowest expression at 21-DAI in the susceptible lines; however, expression pattern of this gene was not consistent with the endogenous level of phenylethylamine in the resistant lines. In the case of tyrosine and tryptophan, both resistant and susceptible lines showed a similar pattern of accumulation over the three time points; however, their accumulation was not consistent with the expression of *AO* (Fig 3C).

### Changes in the profiles of organic acids in *B*. *napus* roots as a result of *P*. *brassicae* infection

**Fig 4:**
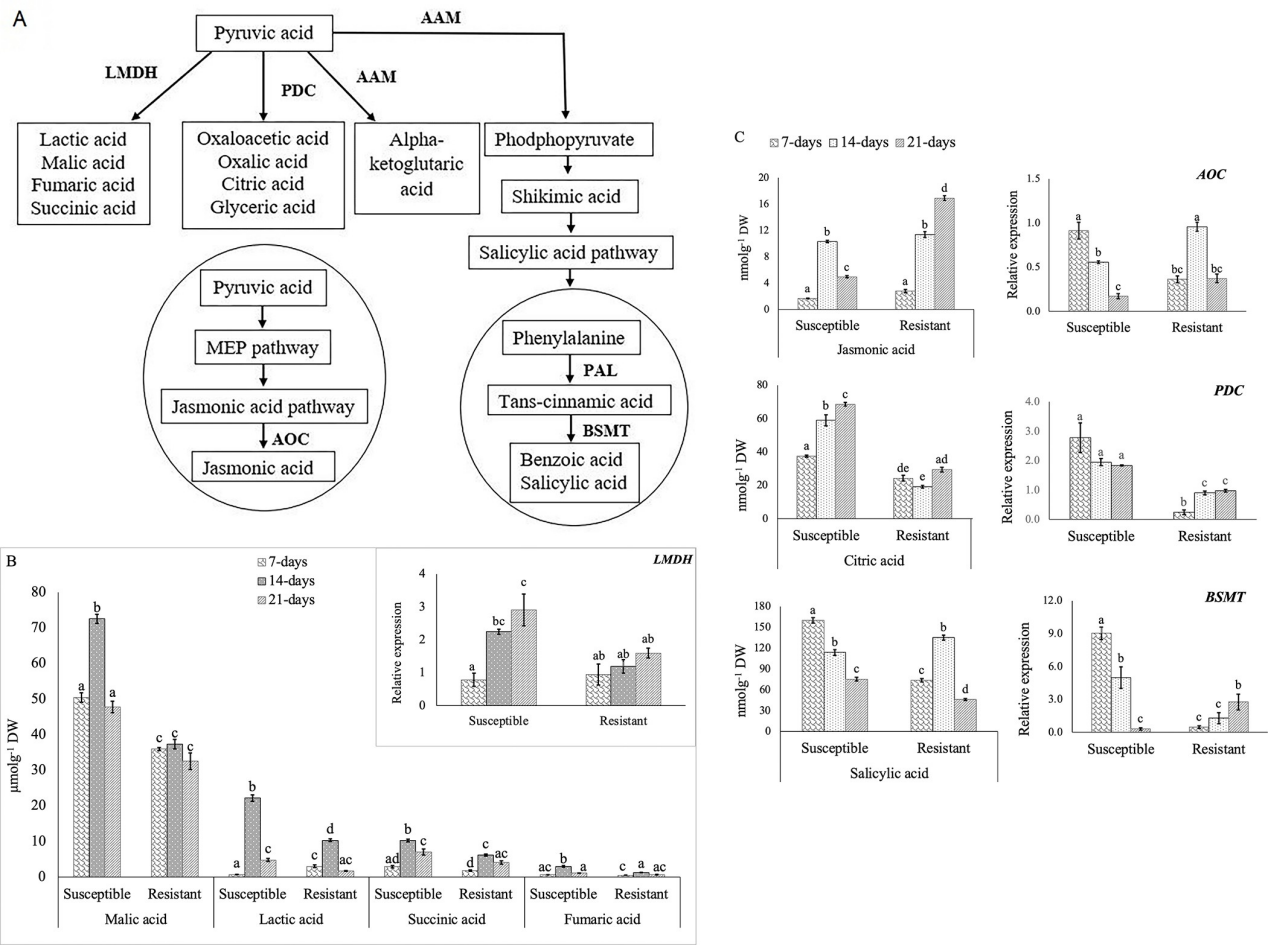
Changes in organic acids and phytohormone profiles in the infected root samples of susceptible and resistant *B*. *napus* canola lines at 7-, 14- and 21-days after inoculation (DAI) with *P*. *brassicae* pathotype 3A. A: Biosynthetic pathways along with the important enzymes involved (bold). B: Accumulation of *LMDH-*leading organic acids and relative expression of *LMDH* (right top corner). C: Accumulation of jasmonic acid (JA), citric acid and salicylic acid (SA) as well as the relative expression of the pathway genes *AOC*, *PDC* and *BSMT*. The gene expression levels were normalized to the corresponding gene expression (1.0) in the control sample i.e. without inoculation.

Organic acids are biosynthesized from a common precursor, pyruvic acid through the actions of several enzymes, represented in Fig 4A. The amount of malic acid significantly increased in the susceptible lines at 14-DAI, and its level at 21-DAI was similar to what was observed at 7-DAI. In the case of the resistant lines, no significant change in the endogenous levels of malic acid was detected over the three time points. The *LMDH* (lactate/malate dehydrogenase)-transcript involved in the biosynthesis of malic acid also showed no significant change in the resistant lines over the three time points (Fig 4B). Nevertheless, we observed a continued increase of *LMDH* transcript in the susceptible lines up to 21-DAI, although the endogenous level of malic acid decreased at this stage. Other organic acids such as lactic, succinic and fumaric acids showed a similar trend of accumulation in both resistant and susceptible lines at 7- to 21-DAI, where the greatest content was detected at 14-DAI (Fig 4B).

The accumulation of jasmonic acid (JA) increased significantly at 14-DAI and decreased at 21-DAI in the susceptible lines, while in the resistant lines, JA increased significantly (*p*-value < 0.05) over the three time points (Fig 4C). However, expression pattern of the gene *AOC* (allene oxide cyclase), postulated to be involved in the biosynthesis of JA, did not correlate with JA content (Fig 4A and 4C). The metabolite citric acid showed an increasing trend of accumulation in the susceptible lines with the progression of infection, while no significant change was observed in the resistant lines (Fig 4C). Overall, the content of citric acid was higher in the susceptible lines as compared to the resistant lines; and the gene coding for *PDC* (pyruvate decarboxylase), which is a part of the pyruvate dehydrogenase (PDH) multienzyme complex that catalyzes the conversion of pyruvate to acetyl coenzyme A, also showed significantly higher expression in the susceptible lines than in the resistant lines (Fig 4C). The amount of salicylic acid (SA) was reduced over the three time points in the susceptible lines (Fig 4C), and this was consistent with a decreased expression of the pathway gene *BSMT* (salicylate/benzoate carboxyl methyltransferase) involved in the biosynthesis of this trans-cinnamic acid, an intermediate in the SA biosynthetic pathway (Fig 4C). In the resistant lines, the content of SA significantly increased at 14-DAI and decreased at 21-DAI; however, expression pattern of *BSMT* was not consistent with the accumulation of this phytohormone (Fig 4C).

### Changes in fatty acid profiles of canola roots as a result of *P*. *brassicae* infection

**Fig 5:**
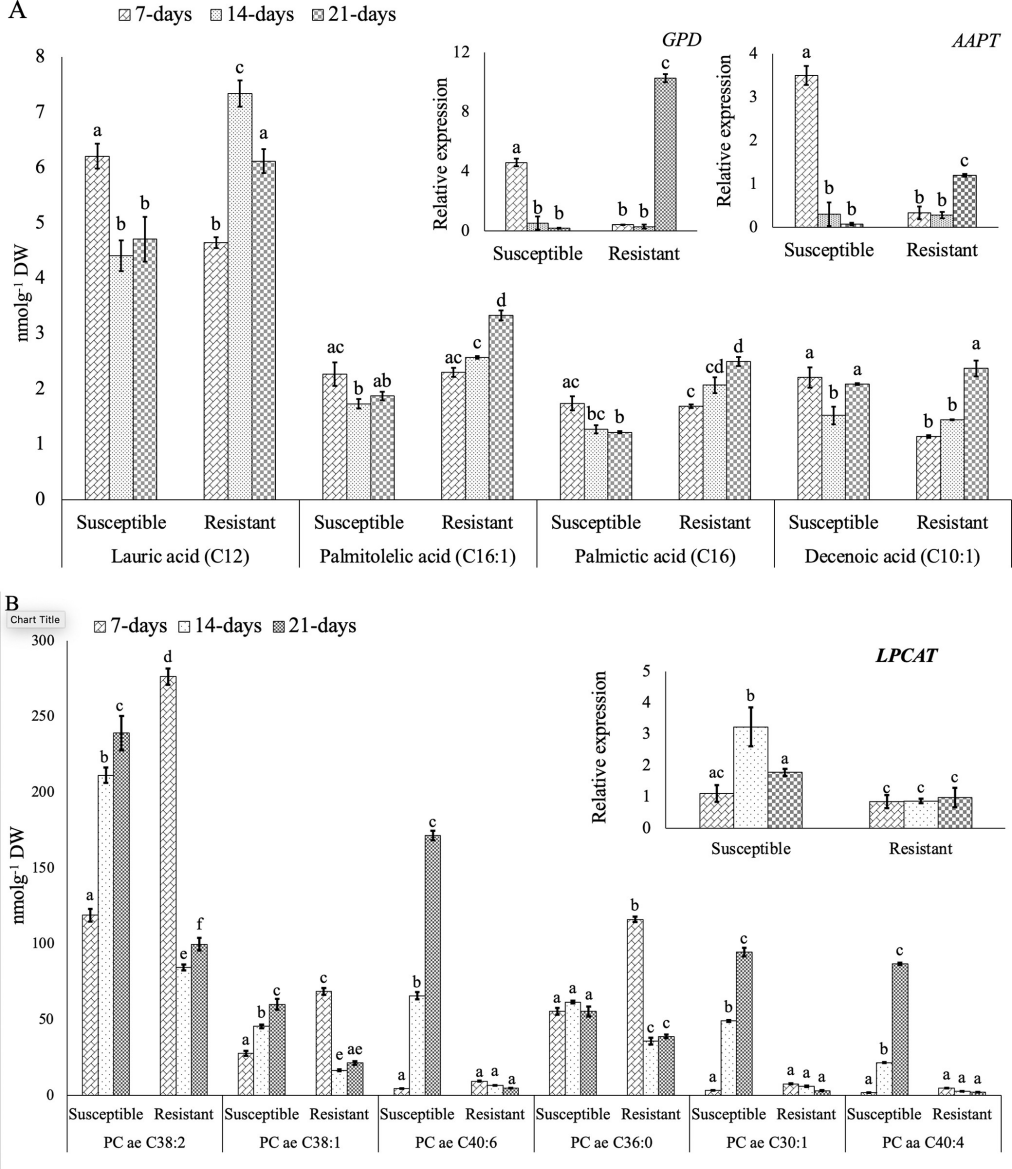
Fatty acid profile of the root samples of the susceptible and resistant *B*. *napus* canola lines inoculated with *P*. *brassicae* pathotype 3A at 7-, 14- and 21-days after inoculation (DAI). A: Accumulation of fatty acids and relative expression of *GPD* and *AAPT*. B: Accumulation of phosphatidylcholines and relative expression of *LPCAT*. Gene expression levels were normalized to the corresponding gene expression (1.0) in the uninoculated controls.

Most of the fatty acids that we profiled showed an increased abundance over the infection period in the resistant lines and exhibited a decreasing trend in the susceptible lines (Fig 5A). Pathway mapping of these fatty acids represented the glycerophospholipid metabolic pathway as the most upregulated pathway in MetPA. The expression of the genes encoding enzymes involved in this pathway, for instance *GPD* and *AAPT*, also correlated with the endogenous levels of these fatty acids over the three time points in the resistant and susceptible lines (Fig 5A).

The endogenous level of most of the phosphatidylcholines significantly (*p-*value < 0.05) increased as the infection progressed in the susceptible lines, while their abundance was either significantly decreased or showed no significant change (*p*-value < 0.05) in the resistant lines. The expression of the pathway gene *LPCAT* (lysophosphatidylcholines acyltransferase) was almost stable in the resistant lines over the three time points whereas, a significantly higher expression of this gene was observed at 14-DAI in the susceptible lines (Fig 5B).

### Effect of salicylic acid (SA) on clubroot disease

**Fig 6:**
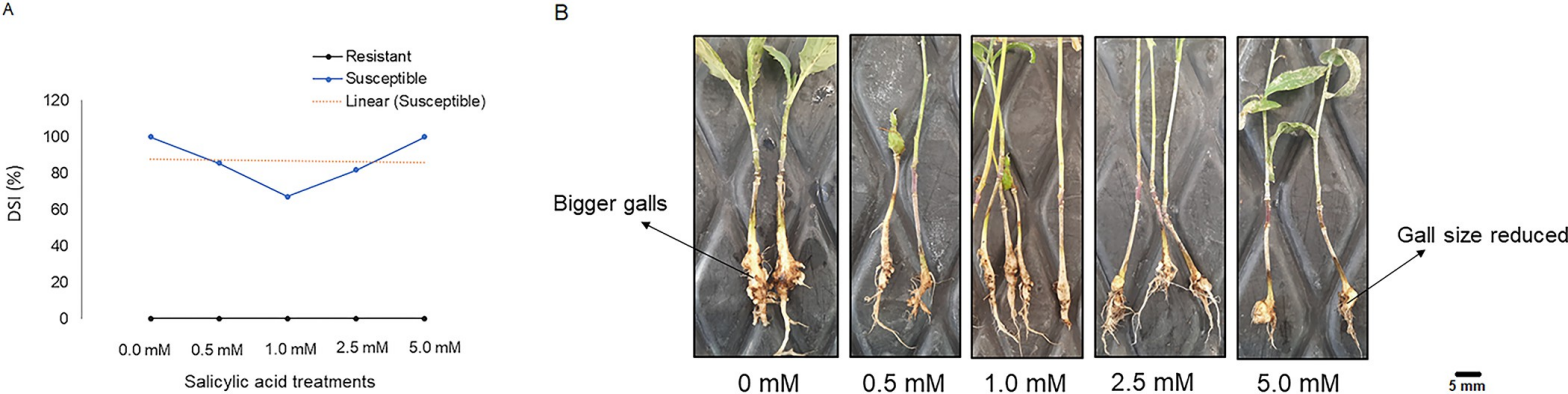
Effects of exogenous application of salicylic acid (SA) on clubroot disease in *B*. *napus*. Exogenous application of SA (0, 0.5, 1.0, 2.5 and 5.0 mM) in susceptible and resistant *B*. *napus* canola lines after inoculation with *P*. *brassicae* pathotype 3A. A: Effect on DSI (%). B: Effect on gall size in the susceptible lines.

In this study (Fig 4C), we observed that the amount of SA was decreased due to the progression of disease in the susceptible lines, while the amount was increased in the resistant lines at 14-DAI, i.e., during the onset of secondary infection. Therefore, we exogenously applied the SA to the roots of the resistant and susceptible *B*. *napus* plants to determine whether such application would have an effect on disease progression. In case of the susceptible lines, a lower concentration of SA (0.5, 1.0 and 2.5 mM) resulted in a reduced disease severity index (DSI) (67 to 81%), while the DSI increased to 100% at a higher concentration (5.0 mM) (Fig 6A). Among the different concentrations, the lowest DSI (67.1%) in the susceptible lines was observed with the 1.0 mM SA treatment, whereas decreased gall sizes were observed at SA concentrations of 0.5 and 1.0 mM (Fig 6B). In case of the resistant lines, no change in DSI (0.0%) was observed due to the application of SA (Fig 6A).

## Discussion

We conducted metabolic profiling of clubroot resistant and susceptible canola lines following inoculation with *P*. *brassicae* pathotype 3A to investigate the changes in primary metabolites that may be involved in the resistance mechanism to this pathogen. The relative endogenous levels of the major polyamines such as putrescine, spermidine and spermine have been profiled in roots of several *Brassica* species [20] and differential expression of the initial pathway genes due to inoculation with *P*. *brassicae* has been reported [21,22]. However, the nature of their accumulation in *B*. *napus* canola due to *P*. *brassicae* infection as well as a combined analysis of these compounds and associated gene expression cannot be found in literature.

In the present study, we observed a stable and lower endogenous level of spermidine and spermine and a stable or elevated expression of the pathway genes *ADC*, *SPDS*, *SPMS* and *SAMDC* in the resistant lines over the course of pathogen infection. In contrast, the content of these two polyamines was higher in the susceptible lines, and their endogenous level and expression of the pathway genes decreased over the progression of pathogen infection (Fig 1B and 1C). This suggests that a higher endogenous level of these polyamines may favor the initial infection; however, they might play a limited role at a later stage of disease development. During the secondary infection in *B*. *napus* at 14- to 21-DAI, the pathogen invades the host root cortex and produces secondary plasmodia causing damage to the host cell wall [23], and this might degrade the cell wall components. Polyamines interact with cell wall components and support cell wall rigidity and cell adhesion [8,9,24]. It has been reported that the endogenous level of putrescine significantly reduces from 14- to 21- DAI in both inoculated and non-inoculated resistant and susceptible *Arabidopsis* [9]. Our study also showed that the endogenous level of spermine and spermidine reduced from 7-DAI to 14-DAI and further to 21-DAI in the susceptible lines. Based on this, it can be postulated that the reduced content of polyamines such as putrescine, spermidine and spermine at 21-DAI, i.e. at the secondary infection stage, might have played a role in cell wall degradation resulting in gall formation in the susceptible *B*. *napus* canola. Hamana et al. (2015) [20] also reported an increased content of polyamines in susceptible lines as compared to resistant lines of *Brassica oleracea* and *B*. *rapa* due to *P*. *brassicae* infection.

The alteration of polyamine content due to pathogen infection was first reported by Greenland and Lewis (1984) [25]. They found an increased content of spermidine in barley leaf due to *Puccinia hordei* infection causing leaf rust disease. Subsequently, several researchers reported an elevated content of polyamines in association with an increased activation of the biosynthetic enzymes such as ADC and ODC due to pathogen infection [for review, see 26]. Walters (2000) [26] summarized several studies, which provided evidence that an increased content of polyamines is associated with the formation of galls or clubbed roots due to *P*. *brassicae* infection in different *Brassica* plants. Polyamines play a major role in cell division; therefore, an increased content of these organic compounds might result in a greater proliferation and differentiation of root cells and that might be associated with clubbed roots in *Brassica*. In contrast, an increased accumulation of spermidine was reported in clubroot resistant line than in susceptible line during *P*. *brassicae* infection in *B*. *rapa* [21].

Jubault et al. (2008) [9] reported a higher expression of the pathway genes *ADC*, *SPDS*, *SPMS* and *SAMDC*, associated with the biosynthesis of polyamines, at 14- and 21- DAI in both susceptible and resistant *Arabidopsis* inoculated with *P*. *brassicae* as compared to the uninoculated ones. An up-regulation of *SPDS* in susceptible *B*. *napus* in response to *P*. *brassicae* infection was also reported by Cao et al. (2008) [27]. Differential expression of the initial pathway gene *ADC* has been reported in *B*. *juncea* following inoculation with *P*. *brassicae* [22]. In the present study, based on accumulation of spermidine and spermine and expression of the associated genes in the susceptible and resistant lines (Fig 1B and 1C), it can be postulated that the expression of the pathway genes might have been upregulated due to pathogen infection. However, gene expression and polyamine contents were not correlated in the resistant lines suggesting that this organic compound may play a complex role in host resistance mechanism. Polyamines have also been reported to provide a crucial physical barrier to pathogen invasion [for review, see 9]. For example, Berta et al. (1997) [24] reported that polyamines are involved in restoring cell wall thickness by strengthening the links between the cell wall components. They are not only found in the plant cell wall but also in cytoplasm, vacuoles, mitochondria, plastids and nuclei in free form. Due to their polycationic nature, they can produce ionic and electrostatic linkages with low molecular weight compounds and proteins of cell components. Hence, they might contribute to the plant resistance mechanism and cell division and differentiation [for review, see 8,20]. Polyamines are also reported to play a major defensive role against environmental stress in plants [for review, see 8,20,22]. Based on the above analysis, it is apparent that further investigation into the role of polyamines in *B*. *napus* canola-*P*. *brassicae* pathosystem will be needed.

The upregulation of primary metabolism to supply the required energy for pathogen development in roots according to susceptibility level was accredited to the biotrophic nature of *P*. *brassicae* [28]. In this study, we observed a higher concentration of amino acids such as serine, glutamic acid, methionine, glycine, alanine, tyrosine, asparagine, glutamine, arginine and proline due to *P*. *brassicae* infection in the susceptible lines as compared to the resistant lines (Fig 2B). Wagner et al. (2012) [10] also found a higher accumulation of asparagine and arginine at a later stage of infection (28- to 35- DAI) in inoculated susceptible *B*. *napus* (disease index 88–95%) as compared to resistant or partial resistant genotypes (disease index 28–50%). Increased amino acid content is characteristic of the establishment of trophic relationship between the host and biotrophic pathogens [29]. The higher accumulation of nitrogen-rich amino acids such as asparagine, glutamine and arginine in susceptible lines could be due to their involvement in nitrogen transport from the host to pathogen-infected tissue [10]; previous study showed that an increased nitrogen supply positively impacts disease development in *B*. *napus* [28]. Wagner et al. (2012) [10] reported a significant correlation between amino acid content and relative pathogen DNA content in *B*. *napus* at a later stage of infection (28- to 35-DAI). In the present study, we also found the highest accumulation of different amino acids at 14-DAI (Fig 2B) in both susceptible and resistant lines; this suggests that their accumulation might be associated with the onset of secondary infection. The association of several amino acids with clubroot susceptibility was also supported by their association with gall formation in susceptible *B*. *napus* [28].

The differential accumulation of amino acids and other metabolites such as phenylethylamine and choline in the susceptible lines due to pathogen infection was more prominent at 14-DAI (Fig 2B). This might be due to the onset of secondary infection in *B*. *napus* that takes place at 12- to 21-DAI depending on host genotype, and this makes the host plant metabolically more active [5,11,30]. In the case of amino acids, Lan et al. (2020) [21] reported that the biosynthesis of phenylalanine and tryptophan significantly increases in clubroot resistant line than in the susceptible *B*. *rapa*. Wagner et al. (2019) [11] also found an association of phenylalanine, threonine, tryptophan and leucine with meta-QTL related to clubroot resistance in *B*. *napus*. During the onset of secondary infection (i.e., 14-DAI), the pathogen disrupts the mitotic cell cycle and the host cell becomes metabolically more active to produce galls that serve as a sink for amino acids [31]. This could be the reason for the higher accumulation of tyrosine and phenylethylamine along with the high expression of *AO* at 14-DAI in the susceptible and resistant lines, respectively (Fig 3C). Conversely, the upregulation of *ASK* at the later stage of infection (21-DAI) led to high glutamine content in the resistant inoculated plants (Fig 3B). Glutamine content was also reported to be associated with resistance in partially resistant (DSI less than 50%) *B*. *napus* [10]. Among the above-mentioned compounds, phenylethylamine, which is derived from phenylalanine and tyrosine, is reported to play a role in plant defense against pathogen infection [32,33]; however, this has not been previously reported in *Brassica*. Therefore, further investigation will be required to confirm the role of phenylethylamine in clubroot disease resistance response.

In this study, relatively higher contents of organic acids were found at 14-DAI in both resistant and susceptible lines. However, on average, the level of these organic acids was higher in the susceptible line (Fig 4B) as compared to the resistant lines; this suggests that they might be involved in clubroot disease susceptibility. Wagner et al. (2019) [11] also found an association of malic and succinic acid contents with disease susceptibility and the QTL involved in the control of these traits are localized in the genomic region where QTL for clubroot susceptibility has been mapped. Aigu et al. (2022) [28] reported that the application of nitrogen fertilizer enhances clubroot gall formation in both resistant and susceptible *B*. *napus* lines, and this was accompanied by a higher accumulation of succinic acid. Organic acid, such as fumaric acid has been also reported to play a role in susceptibility to *Alternaria brassicicola* in Arabidopsis [34]. Thus, our results provided further evidence for the involvement of these organic acids in clubroot disease susceptibility in *B*. *napus* canola.

The contribution of both JA and SA biosynthetic pathways and the expression of related genes in clubroot resistance has been reported by several researchers using the model plant *Arabidopsis* as well as *Brassica* [21,35–37]. For example, JA and SA pathway genes have been reported to be differentially activated due to infection by *P*. *brassicae* in *B*. *rapa* [36,37]. Adhikary et al. (2022) [35] found an upregulation of the SA biosynthetic genes, *isochorismate synthase* and *salicylic acid 3-hydroxylase*, and a downregulation of the JA biosynthetic gene, *jasmonic acid-amido synthetase*, in clubroot resistant *B*. *napus*. Lemarié et al. (2015) [36] found an activation of JA signaling pathway genes at 14-DAI and a decreased expression of these genes at 17-DAI; this correlated well with the content of JA in Arabidopsis. However, inhibition of signaling of these genes was observed at an earlier stage i.e. 10-DAI in both partially resistant and susceptible genotypes. Prerostova et al. (2018) [37] and Irani et al. (2018) [38] reported an elevated expression of *AOC* at a later stage, i.e. at 15- and 24-DAI, in susceptible *B*. *napus* and Arabidopsis, respectively. However, a reduced but moderate activation of *AOC* was also observed at a later stage of infection (15- to 35- DAI) in resistant *B*. *napus* as compared to susceptible genotype [37]. This indicates that the host defense response varies depending on disease progression from primary infection to secondary infection; this was also reported in *Arabidopsis* and *B*. *napus* where JA content and related gene expression varied from early to late stage of infection [36,37]. In this study, we also observed a higher endogenous JA content at 14-DAI as compared to 7-DAI in the susceptible lines (Fig 4C). However, an association of JA with clubroot susceptibility is difficult to explain when JA levels are compared with the expression of *AOC* in the resistant lines over the three time points. When the expression of *AOC* and the endogenous levels of JA in the susceptible and resistant lines at 14-DAI and 21-DAI (Fig 4C) are considered, our results contradict the earlier findings that JA plays a role in clubroot susceptibility during later stages of disease progression. Therefore, further investigation is needed to confirm the role of JA in clubroot susceptibility or resistance in *B*. *napus* canola.

Lan et al. (2020) [21] and Prerostova et al. (2018) [37] reported a higher content of SA in resistant *Brassica* as compared to susceptible genotypes inoculated with *P*. *brassicae* and suggested that SA might play a role in mediating resistance response to this pathogen. In this study, we observed a higher endogenous level of SA in the susceptible lines at 7-DAI as compared to the content observed in the resistant lines. However, SA levels continued to decrease in the susceptible lines with the progression of disease while in the resistant lines, it increased at 14-DAI but significantly decreased at 21-DAI. But the expression of the pathway gene *BSMT* showed an increasing trend over the three time points in the resistant lines (Fig 4C). Ji et al. (2021) [39] reported a significant increase in the amount of SA in susceptible *B*. *rapa* due to pathogen infection; this indicates that pathogen infection may trigger SA production. Lan et al. (2020) [21] detected a higher amount of SA at a later stage (i.e., 28-DAI) of infection in resistant inoculated *B*. *rapa* than in susceptible type as well as in resistant non-inoculated plants. Prerostova et al. (2018) [37] reported a higher content of SA along with higher expression of the pathway genes in resistant *B*. *napus* than in the susceptible one. They also observed that the amount of SA was high at 22-DAI, but significantly decreased at 35-DAI and no trace of this compound was observed at initial infection phage i.e., 2 to 15- DAI [37]. This indicates that the upregulation of SA might be related to secondary infection than at the early or later stage of infection in host resistance mechanism.

The role of SA in clubroot resistance has also been demonstrated by several researchers through exogenous application of this compound. For example, Xi et al. (2021) [40] treated *P*. *brassicae-*inoculated susceptible *B*. *rapa* with 0.06 mM salicylic acid and found a significantly lower DSI (25.51%) when compared with the non-treated plants (66.15%). Another study on exogenous application of SA in susceptible *B*. *oleracea* inoculated with *P*. *brassicae* showed that a concentration of 1.0 mM to 5 mM reduces gall formation and disease severity, where the 1.0 mM SA treatment was the most effective and reduced gall size [41]. Similar effectiveness of lower SA concentration (i.e., 1 mM and 2 mM) was also reported by Prerostova et al. (2018) [37], where they found a decrease in disease severity from 100 to 19% and 40 to 3.7% DSI in susceptible and partially resistant *B*. *napus*, respectively. In this study, we also observed that exogenous application of SA (0.5 to 2.5 mM) reduces DSI (%) and gall size in susceptible *B*. *napus* at 35 days after SA treatment (Fig 6A and 6B), where the 1.0 mM treatment resulted in the greatest reduction of DSI (100% to 67.1%) and gall size (Fig 6A and 6B). A concentration of SA above 10 mM can result in reduced plant growth or plant mortality [39,41]. This further confirms that SA inhibits gall formation and reduces disease severity; however, its effect varies depending on the concentration; a lower concentration (0.5 to 2.5 mM) is recommended to combat this disease. Citric acid has been reported as the susceptibility marker in *Brassica* [11,21]. In this study, we also found a higher accumulation of citric acid with the progression of pathogen infection in the susceptible lines (Fig 4C).

The significant upregulation of *GPD* and *AAPT* leading to a higher accumulation of fatty acids, such as lauric, palmitolelic, palmictic and decenoic acid, at 14- and 21-DAI in clubroot resistant canola supported their role in host-resistance mechanism (Fig 5A). Previously, researchers have reported increased contents of decenoic and palmictic acid and upregulation of the associated lipid biosynthetic genes at a later stage of infection in *P*. *brassicae-*inoculated resistant *B*. *rapa* (28-DAI) and *Arabidopsis* (17- to 20-DAI) [21,38]. This suggests that the resistant genotypes accumulate and biosynthesize higher amounts of these fatty acids. An increased accumulation of fatty acids with the progression of disease in the resistant lines could be due to a higher expression of the pathway genes, as we observed in this study, or due to transportation of lipids or fatty acids to the sites of infection [42]. Hence, the elevated content of these metabolites may provide a defense response in the resistant lines.

The fatty acid derivatives, such as phosphatidylcholines are the major compound of the fungal membrane [43]. We found a significantly decreased amount of phosphatidylcholines at 14- and 21-DAI, i.e. during secondary infection, as compared to the amount detected at the early stage of infection (7-DAI) in the resistant lines as compared to the susceptible lines (Fig 5B). Leakage of these cytoplasmic substances causes fungal cell membrane disorganization and reduces the effectivity of the pathogen to progress the infection [44,45]. In this regard, the decreased amount of these phosphatidylcholines found in the resistant lines with disease progression might be associated with host resistance mechanism. This was also evident from the observed opposite trend of accumulation of most of the phosphatidylcholines in the susceptible line (increased content with disease progression).

## Conclusion

In this study, we demonstrated the role of different primary metabolites viz. polyamines, amino acids, organic acids, fatty acids and their derivatives at different stages of infection in clubroot disease resistance or susceptibility. This result has been further substantiated through expression analysis of the genes involved in the biosynthesis of these metabolites. The findings from this integrated study based on metabolomic and gene expression analysis provide an in-depth knowledge of host-pathogen interactions in terms of primary metabolism and their regulation, and the putative genes and pathways that trigger the host resistance mechanism to clubroot disease in canola. However, the gene expression patterns did not always show a correlation with the accumulation of metabolite over the progression of the disease. One of the reasons for this could be that we focused on the rate-limiting enzymes leading to the accumulation of these metabolites; studying the branch pathway genes leading to the biosynthesis of these metabolites may further extend our knowledge in this regard.

## Supporting information

S1 Table The list of primers designed based on the transcripts involved in polyamines, amino acids, organic acids and fatty acids biosynthetic pathways in plants. (DOCX)

S2 Table The concentration of polyamines (nmolg^-1^ DW) in non-infected (control) samples of susceptible and resistant lines of *Brassica napus* at different days after inoculation (SE = Standard error). (DOCX)

S3 Table ANOVA post-hoc analysis results of detected amino acids in infected with *Plasmodiophora brassicae* and non-infected root samples of clubroot resistant and susceptible *Brassica napus* canola lines collected at 7, 14 and 21 days after inoculation/non-inoculation (control). (DOCX)

S4 Table Pathways retrieved from MetPA (MetaboAnalyst 5.0) for amino acids detected in *Brassica napus* canola root samples (Total, the total number of metabolites involved in each pathway in KEGG; Hits, number of metabolites matched from sample data; Impact, pathway impact). (DOCX)

S5 Table Concentrations (nmolg^-1^DW) of fatty acids and their derivatives (lysophosphatidylcholines and phosphatidylcholines) in infected susceptible and resistant *Brassica napus* canola lines with *Plasmodiophora brassicae*. (DOCX)
