# Supplementary material for: Changes in primary metabolism and associated gene expression during host-pathogen interaction in clubroot resistance of Brassica napus
Source: PLoS One. 2024 Sep 9;19(9):e0310126. doi: 10.1371/journal.pone.0310126 (PMC11383247; doi:10.1371/journal.pone.0310126)
Supplement: S1 Table — (DOCX) [file pone.0310126.s001.docx]

**Supplementary tables:**

**S1 Table**

| **Primers** | **Primer sequence** | **Product size (bp)** | ***Arabidopsis* homolog** | ***Brassica napus* homolog chromosome and region** |
| --- | --- | --- | --- | --- |
| UBC10-F | GTCCTTACGCTGGTGGTGTT |  |  |  |
| UBC10-R | TGGAGATGGTGAGAGCAGGA |  |  |  |
| ADC-F | ATCGAGCAGTTGGCTTCTGT | 208 | At2g16500 | chrA08: 10129446 to 10131530 |
| ADC-R | ACGTCAAATCCGACAAGACC |  |  |  |
| SPDS-F | AGAGTGTGCCTCTCCTGCAT | 246 | At1g23820 | chrA08: 15357177 to 15359227 |
| SPDS-R | CCAGAGAAGCCTCTGTCACC |  |  |  |
| SPMS**-**F | AGAGTGTGCCTCTCCTGCAT | 246 | At5g53120 | chrA08: 15357177 to 15359141 |
| SPMS-R | CCAGAGAAGCCTCTGTCACC |  |  |  |
| SAMDC-F | GCGACTGCAACAACAATGTC | 99 | At3g02470 | chrA03: 13680435 to13681538 |
| SAMDC-R | TCTCCCCACCAGTTTCATTC |  |  |  |
| ASK-F | CACCAATATGCACCATCTCG | 101 | At1g31230 | chrA09: 18380820 to 18385057 |
| ASK-R | TGCTACCGCATCAACAACTC |  |  |  |
| ARG-F | TCCTTGTTTGCTGTGTCAGG | 138 | At4g08900 | chrA03: 11411562 to 11413063 |
| ARG-R | TCAGGATGTGCATCAAGGTG |  |  |  |
| AO-F | GAGGAAGCTTGCTTGCTTTG | 120 | At4g14940 | chrC01: 16170671 to 16174562 |
| AO-R | AAACCCACGAGCTCATCATC |  |  |  |
| SHM-F | TCGAACGGTGAAGGGATTAC | 96 | At4g37930 | chrA08: 13152554 to 13154405 |
| SHM-R | TTGGCAGGTGTGTAACAAGC |  |  |  |
| iIVE-F | ATTTACGTGTCTCCCGTTGG | 133 | At3g49680 | chrA08: 1821950 to 1822928 |
| iIVE-R | AAGCCTGAGCTCACCTAAACAC |  |  |  |
| PDC-F | AATGTGGGATGAGGAGTTGC | 85 | At3g49680 | chrC01: 37972791 to 37974311 |
| PDC-R | AGGCTGTATCGATGGCTTTG |  |  |  |
| LMDH-F | ATGAGTGGTTTCCCCAGATG | 102 | At5g43330 | chrA08: 18443389 to 18445442 |
| LMDH-R | TGACAAGGCTTGACCACAAC |  |  |  |
| AAM-F | GGGAACACAAAGCAAGAACC | 135 | At1g17290 | chrA09: 30879075 to 30881945 |
| AAM-R | TTGGCCAACAGCCTATAACC |  |  |  |
| BSMT-F | CGGACAGAACTCATTTGTGG | 117 | At3g11480 | chrA03: 15305871 to 15308994 |
| BSMT-R | TTCTCGGGGAGATCATTCAG |  |  |  |
| PAL-F | TATGGCCGCGATAGCTAAAC | 124 | At2g37040 | chrA04: 16185037 to 16187792 |
| PAL-R | AAACTCCGTCAACGACAACC |  |  |  |
| AOC-F | TGAAGCTTCGTCAGCTTGTG | 85 | At1g13280 | chrA09: 12631513 to 12632365 |
| AOC-R | GTAAGCTCCAACGGCAAATC |  |  |  |
| GPD-F | ATATCCCACCGTTGCTTCAC | 87 | At5g40610 | chrA04: 9171928 to 9173890 |
| GPD-R | CTGGCTTCACATTTGCTGTG |  |  |  |
| AAPT-F | CTTGTGCGGTATGCATTGAC | 126 | At1g13560 | chrA09: 31451140 to 31453873 |
| AAPT-R | CATAGCAGTGCTTCCAAACG |  |  |  |
| LPCAT-F | ATGACCATTGGCTACGCTTC | 73 | At1g63050 | chrA09: 6959862 to 6961920 |
| LPCAT-R | CGAAACCGAGGAAGAAAGTG |  |  |  |
| PGK-F | CTCTTCTTCGCCATGGAAAC | 149 | At3g12780 | chrA03: 15641705 to 15644187 |
| PGK-R | ATCACCCCCTCCTCAAAATC |  |  |  |

F, Forward (5' to 3'); R, Reverse (3' to 5')
