# Supplementary material for: Changes in primary metabolism and associated gene expression during host-pathogen interaction in clubroot resistance of Brassica napus
Source: PLoS One. 2024 Sep 9;19(9):e0310126. doi: 10.1371/journal.pone.0310126 (PMC11383247; doi:10.1371/journal.pone.0310126)
Supplement: S2 Table — (DOCX) [file pone.0310126.s002.docx]

**S2 Table**

| **Polyamines** | **Roots (control)** | **7-days** | **14-days** | **21-days** | **Mean ± SE** | ***p*-value** |
| --- | --- | --- | --- | --- | --- | --- |
| Putrescine | Susceptible | 199.56 | 73.06 | 102.05 | 124.89 ± 38.26 | 0.52 |
|  | Resistant | 101.07 | 110.82 | 72.06 | 94.65 ± 11.64 |  |
| Spermidine | Susceptible | 80.52 | 9.17 | 14.83 | 34.84 ± 22.90 | 0.79 |
|  | Resistant | 55.52 | 20.73 | 4.87 | 27.04 ± 14.96 |  |
| Spermine | Susceptible | 6.53 | 3.48 | 3.53 | 4.51 ± 1.01 | 0.47 |
|  | Resistant | 3.88 | 3.97 | 2.95 | 3.60 ± 0.33 |  |
