# Supplementary material for: Changes in primary metabolism and associated gene expression during host-pathogen interaction in clubroot resistance of Brassica napus
Source: PLoS One. 2024 Sep 9;19(9):e0310126. doi: 10.1371/journal.pone.0310126 (PMC11383247; doi:10.1371/journal.pone.0310126)
Supplement: S3 Table — (DOCX) [file pone.0310126.s003.docx]

**S3 Table**

| **Amino acids** | ***p*-value** | **Fisher's LSD** |
| --- | --- | --- |
| Phenylethylamine | 4.43E-10 | 14CS - 14CR; 14IR - 14CR; 14CR - 21IR; 14CR - 21IS; 7CR - 14CR; 7CS - 14CR; 7IS - 14CR; 14IR - 14CS; 14CS - 14IS; 14CS - 21CR; 14CS - 21CS; 14CS - 21IR; 14CS - 21IS; 7CR - 14CS; 14CS - 7IR; 14IR - 14IS; 14IR - 21CR; 14IR - 21CS; 14IR - 21IR; 14IR - 21IS; 14IR - 7IR; 14IS - 21CR; 14IS - 21CS; 14IS - 21IR; 14IS - 21IS; 7CR - 14IS; 7CS - 14IS; 7IS - 14IS; 7CR - 21CR; 7CS - 21CR; 7IS - 21CR; 7CR - 21CS; 7CS - 21CS; 7IS - 21CS; 7CR - 21IR; 7CS - 21IR; 7IR - 21IR; 7IS - 21IR; 7CR - 21IS; 7CS - 21IS; 7IR - 21IS; 7IS - 21IS; 7CR - 7IR; 7CS - 7IR; 7IS - 7IR |
| Alanine | 2.71E-08 | 14IS - 14CR; 7CS - 14CR; 7IS - 14CR; 14IS - 14CS; 7CR - 14CS; 7CS - 14CS; 7IR - 14CS; 7IS - 14CS; 14IS - 14IR; 7CS - 14IR; 14IS - 21CR; 14IS - 21CS; 14IS - 21IR; 14IS - 21IS; 14IS - 7CR; 14IS - 7IR; 14IS - 7IS; 7CR - 21CR; 7CS - 21CR; 7IR - 21CR; 7IS - 21CR; 7CR - 21CS; 7CS - 21CS; 7IR - 21CS; 7IS - 21CS; 7CS - 21IR; 7IR - 21IR; 7IS - 21IR; 7CS - 21IS; 7CS - 7CR; 7CS - 7IR; 7CS - 7IS |
| Glycine | 3.08E-08 | 14IS - 14CR; 7CS - 14CR; 7IS - 14CR; 14IR - 14CS; 14IS - 14CS; 7CR - 14CS; 7CS - 14CS; 7IR - 14CS; 7IS - 14CS; 14IS - 14IR; 14IR - 21CR; 14IR - 21CS; 7CS - 14IR; 7IS - 14IR; 14IS - 21CR; 14IS - 21CS; 14IS - 21IR; 14IS - 21IS; 14IS - 7CR; 14IS - 7IR; 14IS - 7IS; 7CR - 21CR; 7CS - 21CR; 7IR - 21CR; 7IS - 21CR; 7CR - 21CS; 7CS - 21CS; 7IR - 21CS; 7IS - 21CS; 7CR - 21IR; 7CS - 21IR; 7IS - 21IR; 7CS - 21IS; 7IS - 21IS; 7CS - 7CR; 7CS - 7IR; 7CS - 7IS; 7IS - 7IR |
| Proline | 1.84E-07 | 14CS - 14CR; 14IR - 14CR; 14IS - 14CR; 14CR - 21CR; 14CR - 21CS; 14CR - 21IR; 7CS - 14CR; 14CS - 21CR; 14CS - 21CS; 14CS - 21IR; 14CS - 21IS; 7CS - 14CS; 14IR - 21CR; 14IR - 21CS; 14IR - 21IR; 14IR - 21IS; 14IR - 7IR; 14IS - 21CR; 14IS - 21CS; 14IS - 21IR; 14IS - 21IS; 14IS - 7IR; 21IS - 21CR; 7CR - 21CR; 7CS - 21CR; 7IR - 21CR; 7IS - 21CR; 7CR - 21CS; 7CS - 21CS; 7IR - 21CS; 7IS - 21CS; 7CR - 21IR; 7CS - 21IR; 7IR - 21IR; 7IS - 21IR; 7CR - 21IS; 7CS - 21IS; 7IS - 21IS; 7CS - 7CR; 7CS - 7IR; 7CS - 7IS |
| Serine | 2.88E-07 | 14IS - 14CR; 21IS - 14CR; 7CR - 14CR; 7CS - 14CR; 7IS - 14CR; 14IS - 14CS; 21IS - 14CS; 7CR - 14CS; 7CS - 14CS; 7IS - 14CS; 14IS - 14IR; 14IR - 21CR; 21IS - 14IR; 7CS - 14IR; 14IS - 21CR; 14IS - 21CS; 14IS - 21IR; 14IS - 7CR; 14IS - 7CS; 14IS - 7IR; 14IS - 7IS; 21IS - 21CR; 7CR - 21CR; 7CS - 21CR; 7IS - 21CR; 21IS - 21CS; 7CR - 21CS; 7CS - 21CS; 7IS - 21CS; 21IS - 21IR; 7CR - 21IR; 7CS - 21IR; 7IS - 21IR; 21IS - 7CR; 21IS - 7IR; 21IS - 7IS; 7CR - 7IR; 7CS - 7IR; 7IS - 7IR |
| Valine | 3.64E-07 | 14CR - 21CR; 14CR - 21CS; 14CR - 21IR; 7CR - 14CR; 7CS - 14CR; 14IS - 14CS; 14CS - 21CR; 14CS - 21CS; 14CS - 21IR; 7CR - 14CS; 7CS - 14CS; 7IS - 14CS; 14IR - 21CR; 14IR - 21CS; 14IR - 21IR; 7CR - 14IR; 7CS - 14IR; 7IS - 14IR; 14IS - 21CR; 14IS - 21CS; 14IS - 21IR; 14IS - 21IS; 14IS - 7IR; 21IS - 21CR; 7CR - 21CR; 7CS - 21CR; 7IR - 21CR; 7IS - 21CR; 21IS - 21CS; 7CR - 21CS; 7CS - 21CS; 7IR - 21CS; 7IS - 21CS; 21IS - 21IR; 7CR - 21IR; 7CS - 21IR; 7IR - 21IR; 7IS - 21IR; 7CR - 21IS; 7CS - 21IS; 7IS - 21IS; 7CR - 7IR; 7CS - 7IR; 7IS - 7IR |
| Tyrosine | 3.25E-06 | 14IS - 14CR; 7CR - 14CR; 7CS - 14CR; 7IS - 14CR; 14IS - 14CS; 14CS - 21CR; 14CS - 21IR; 7CS - 14CS; 14IS - 14IR; 14IR - 21CR; 14IR - 21CS; 14IR - 21IR; 14IR - 21IS; 14IS - 21CR; 14IS - 21CS; 14IS - 21IR; 14IS - 21IS; 14IS - 7CR; 14IS - 7IR; 14IS - 7IS; 7CR - 21CR; 7CS - 21CR; 7IR - 21CR; 7IS - 21CR; 7CR - 21CS; 7CS - 21CS; 7IS - 21CS; 7CR - 21IR; 7CS - 21IR; 7IR - 21IR; 7IS - 21IR; 7CR - 21IS; 7CS - 21IS; 7IS - 21IS; 7CS - 7IR |
| Glutamic acid | 3.79E-06 | 14IS - 14CR; 21IS - 14CR; 7CS - 14CR; 7IS - 14CR; 14IS - 14CS; 21IS - 14CS; 7CR - 14CS; 7CS - 14CS; 7IR - 14CS; 7IS - 14CS; 14IS - 14IR; 21IS - 14IR; 7CS - 14IR; 7IS - 14IR; 14IS - 21CR; 14IS - 21CS; 14IS - 21IR; 14IS - 7CR; 14IS - 7CS; 14IS - 7IR; 14IS - 7IS; 21IS - 21CR; 7CS - 21CR; 7IS - 21CR; 21IS - 21CS; 7CS - 21CS; 7IS - 21CS; 21IS - 21IR; 7IS - 21IR; 21IS - 7CR; 21IS - 7CS; 21IS - 7IR; 21IS - 7IS |
| Isoleucine | 3.95E-06 | 14CR - 21CR; 14CR - 21CS; 14CR - 21IR; 7CR - 14CR; 7CS - 14CR; 7IS - 14CR; 14CS - 21CR; 14CS - 21CS; 14CS - 21IR; 7CR - 14CS; 7CS - 14CS; 7IS - 14CS; 14IR - 21CR; 14IR - 21CS; 14IR - 21IR; 7CR - 14IR; 7CS - 14IR; 7IS - 14IR; 14IS - 21CR; 14IS - 21CS; 14IS - 21IR; 7CS - 14IS; 7CR - 21CR; 7CS - 21CR; 7IR - 21CR; 7IS - 21CR; 7CR - 21CS; 7CS - 21CS; 7IR - 21CS; 7IS - 21CS; 7CR - 21IR; 7CS - 21IR; 7IR - 21IR; 7IS - 21IR; 7CR - 21IS; 7CS - 21IS; 7IS - 21IS; 7CS - 7IR |
| Homoserine | 4.98E-06 | 14IS - 14CR; 14CR - 21IR; 7CR - 14CR; 7CS - 14CR; 7IS - 14CR; 14IS - 14CS; 14CS - 21CR; 14CS - 21IR; 7CR - 14CS; 7CS - 14CS; 7IS - 14CS; 14IR - 21CR; 14IR - 21CS; 14IR - 21IR; 7CR - 14IR; 7CS - 14IR; 7IS - 14IR; 14IS - 21CR; 14IS - 21CS; 14IS - 21IR; 14IS - 21IS; 7CR - 21CR; 7CS - 21CR; 7IR - 21CR; 7IS - 21CR; 7CR - 21CS; 7CS - 21CS; 7IR - 21CS; 7IS - 21CS; 7CR - 21IR; 7CS - 21IR; 7IR - 21IR; 7IS - 21IR; 7CR - 21IS; 7CS - 21IS; 7IS - 21IS |
| Arginine | 1.47E-05 | 14IR - 14CR; 7CS - 14CR; 7IS - 14CR; 14IR - 14CS; 14IS - 14CS; 7CR - 14CS; 7CS - 14CS; 7IS - 14CS; 14IR - 14IS; 14IR - 21CR; 14IR - 21CS; 14IR - 21IR; 14IR - 21IS; 14IR - 7CR; 14IR - 7CS; 14IR - 7IR; 14IR - 7IS; 14IS - 21CR; 14IS - 21CS; 14IS - 21IR; 14IS - 21IS; 7CR - 21CR; 7CS - 21CR; 7IS - 21CR; 7CR - 21CS; 7CS - 21CS; 7IS - 21CS; 7CR - 21IR; 7CS - 21IR; 7IS - 21IR; 7CR - 21IS; 7CS - 21IS; 7IS - 21IS; 7CS - 7IR |
| Phenylalanine | 4.25E-05 | 14IR - 14CR; 14IS - 14CR; 21IR - 14CR; 21IS - 14CR; 7CR - 14CR; 7CS - 14CR; 7IS - 14CR; 14IS - 14CS; 14CS - 21CR; 21IR - 14CS; 21IS - 14CS; 7CR - 14CS; 7CS - 14CS; 14IR - 21CR; 14IR - 21CS; 14IS - 21CR; 14IS - 21CS; 21IR - 21CR; 21IS - 21CR; 7CR - 21CR; 7CS - 21CR; 7IR - 21CR; 7IS - 21CR; 21IR - 21CS; 21IS - 21CS; 7CR - 21CS; 7CS - 21CS; 7IS - 21CS; 7CR - 7IR; 7CS - 7IR |
| Choline | 6.49E-05 | 14IS - 14CR; 7CS - 14CR; 14IS - 14CS; 7CS - 14CS; 14IS - 14IR; 14IR - 21CR; 14IR - 21CS; 14IR - 21IR; 14IS - 21CR; 14IS - 21CS; 14IS - 21IR; 14IS - 21IS; 14IS - 7CR; 14IS - 7CS; 14IS - 7IR; 14IS - 7IS; 21IS - 21CR; 7CR - 21CR; 7CS - 21CR; 7IS - 21CR; 21IS - 21CS; 7CR - 21CS; 7CS - 21CS; 7IS - 21CS; 21IS - 21IR; 7CR - 21IR; 7CS - 21IR; 7IS - 21IR; 7CS - 7IR |
| Asparagine | 0.0001 | 7CR - 14CR; 7CS - 14CR; 7IS - 14CR; 14IS - 14CS; 7CR - 14CS; 7CS - 14CS; 7IS - 14CS; 7CR - 14IR; 7CS - 14IR; 7IS - 14IR; 14IS - 21CR; 14IS - 21CS; 14IS - 21IR; 21IS - 21CR; 7CR - 21CR; 7CS - 21CR; 7IR - 21CR; 7IS - 21CR; 21IS - 21CS; 7CR - 21CS; 7CS - 21CS; 7IR - 21CS; 7IS - 21CS; 7CR - 21IR; 7CS - 21IR; 7IS - 21IR; 7CS - 21IS; 7IS - 21IS; 7CR - 7IR; 7CS - 7IR; 7IS - 7IR |
| Glutamine | 0.000339 | 7CR - 14CR; 7IS - 14CR; 7CR - 14CS; 7CS - 14CS; 7IS - 14CS; 7CR - 14IR; 7IS - 14IR; 7IS - 14IS; 7CR - 21CR; 7CS - 21CR; 7IS - 21CR; 7CR - 21CS; 7IS - 21CS; 21IR - 21IS; 7IS - 21IR; 7CR - 21IS; 7CS - 21IS; 7IS - 21IS; 7CR - 7IR; 7IS - 7CS; 7IS - 7IR |
| Tryptophan | 0.001405 | 14CR - 21CR; 14CR - 21CS; 14CR - 21IR; 14CR - 21IS; 14CS - 21CR; 14CS - 21CS; 14CS - 21IR; 14CS - 21IS; 14IR - 21CR; 14IR - 21CS; 14IR - 21IR; 14IR - 21IS; 14IS - 21CR; 14IS - 21CS; 14IS - 21IR; 14IS - 21IS; 7CR - 21CR; 7CS - 21CR; 7IR - 21CR; 7IS - 21CR; 7CS - 21CS; 7CS - 21IR; 7CS - 21IS |
| Leucine | 0.00247 | 14CR - 21CR; 14IR - 21CR; 14IR - 21CS; 14IR - 21IR; 14IR - 21IS; 14IS - 21CR; 14IS - 21CS; 14IS - 21IR; 7CR - 21CR; 7CS - 21CR; 7IR - 21CR; 7IS - 21CR; 7CR - 21CS; 7CS - 21CS; 7IR - 21CS; 7IS - 21CS; 7CR - 21IR; 7CS - 21IR; 7IR - 21IR; 7IS - 21IR; 7CS - 21IS; 7IR - 21IS |
| Methionine | 0.005467 | 14IS - 14CR; 7IR - 14CR; 14IS - 14CS; 7CS - 14CS; 7IR - 14CS; 14IS - 14IR; 7IR - 14IR; 14IS - 21CR; 14IS - 21CS; 14IS - 21IR; 14IS - 21IS; 7CS - 21CR; 7IR - 21CR; 7IS - 21CR; 7CS - 21CS; 7IR - 21CS; 7CS - 21IR; 7IR - 21IR; 7IS - 21IR; 7CS - 21IS; 7IR - 21IS; 7IS - 21IS |
| Threonine | 0.00921 | 14CS - 21CR; 14CS - 21CS; 14CS - 21IR; 14IR - 21CR; 14IR - 21CS; 14IR - 21IR; 14IR - 21IS; 14IS - 21CR; 14IS - 21CS; 14IS - 21IR; 7CR - 21CR; 7CS - 21CR; 7IS - 21CR; 7CR - 21CS; 7CS - 21CS; 7IS - 21CS; 7CR - 21IR; 7CS - 21IR; 7IS - 21IR; 7CR - 21IS |

IR=Infected Resistant bulk; CR=Control Resistant bulk; IS= Infected Susceptible bulk; CS= Control Susceptible bulk; 7, 14 and 21= days after inoculation/non-inoculation (control).
