# Supplementary material for: Changes in primary metabolism and associated gene expression during host-pathogen interaction in clubroot resistance of Brassica napus
Source: PLoS One. 2024 Sep 9;19(9):e0310126. doi: 10.1371/journal.pone.0310126 (PMC11383247; doi:10.1371/journal.pone.0310126)
Supplement: S4 Table — (DOCX) [file pone.0310126.s004.docx]

**S4 Table**

| **No.** | **Suggested pathways** | **Total** | **Hits** | ***p*-value** | **Impact** |
| --- | --- | --- | --- | --- | --- |
| 1 | Glycine, serine and threonine metabolism | 33 | 5 | 0.00002 | 0.398 |
| 2 | Valine, leucine and isoleucine biosynthesis | 22 | 4 | 0.00009 | 0.011 |
| 3 | Alanine, aspartate and glutamate metabolism | 22 | 4 | 0.00009 | 0.518 |
| 4 | Arginine biosynthesis | 18 | 3 | 0.001 | 0.169 |
| 5 | Phenylalanine, tyrosine and tryptophan biosynthesis | 22 | 3 | 0.002 | 0.022 |
| 6 | Arginine and proline metabolism | 34 | 3 | 0.007 | 0.252 |
