# Supplementary material for: Changes in primary metabolism and associated gene expression during host-pathogen interaction in clubroot resistance of Brassica napus
Source: PLoS One. 2024 Sep 9;19(9):e0310126. doi: 10.1371/journal.pone.0310126 (PMC11383247; doi:10.1371/journal.pone.0310126)
Supplement: S5 Table — (DOCX) [file pone.0310126.s005.docx]

**S5 Table**

| **Fatty acids and their derivatives** | **Infected Susceptible** | | | **Infected Resistant** | | |
| --- | --- | --- | --- | --- | --- | --- |
|  | **7-days** | **14-days** | **21-days** | **7-days** | **14-days** | **21-days** |
| **Fatty acids** | | | | | | |
| **C4** | 0.645 | 0.599 | 0.826 | 0.526 | 0.704 | 0.676 |
| **C3OH** | 0.833 | 0.887 | 0.730 | 0.763 | 0.794 | 0.820 |
| **C5:1** | 0.766 | 0.616 | 0.725 | 0.657 | 0.859 | 0.735 |
| **C5** | 0.843 | 0.941 | 0.915 | 0.827 | 1.245 | 0.984 |
| **C4OH** | 0.700 | 1.726 | 1.308 | 0.930 | 0.704 | 0.533 |
| **C6:1** | 0.351 | 0.482 | 0.454 | 0.363 | 0.501 | 0.570 |
| **C6** | 0.847 | 1.004 | 1.069 | 0.811 | 0.980 | 1.002 |
| **C8** | 0.554 | 0.498 | 0.402 | 0.365 | 0.409 | 0.465 |
| **C5MDC** | 0.720 | 0.854 | 0.823 | 0.730 | 0.716 | 0.835 |
| **C10:2** | 1.037 | 0.916 | 0.902 | 0.816 | 0.881 | 0.786 |
| **C10** | 1.002 | 1.189 | 1.222 | 1.028 | 1.152 | 1.124 |
| **C12:1** | 1.915 | 1.814 | 2.148 | 1.026 | 1.822 | 1.959 |
| **C14:2** | 0.968 | 1.704 | 1.511 | 0.734 | 0.899 | 0.687 |
| **C14** | 0.622 | 0.446 | 0.398 | 0.390 | 0.371 | 0.477 |
| **C18** | 0.429 | 0.362 | 0.496 | 0.305 | 0.396 | 0.372 |
| **Lysophosphatidylcholines** | | | | | | |
| **LysoPC a C16:0** | 6.892 | 4.203 | 2.020 | 5.309 | 4.741 | 2.671 |
| **LysoPC a C18:2** | 4.151 | 5.533 | 2.301 | 2.663 | 4.071 | 2.818 |
| **LysoPC a C18:1** | 3.693 | 3.994 | 2.011 | 2.307 | 3.480 | 1.879 |
| **LysoPC a C18:0** | 1.472 | 1.176 | 0.536 | 1.871 | 1.191 | 1.042 |
| **LysoPC a C20:3** | 0.519 | 0.560 | 0.557 | 0.522 | 0.496 | 0.487 |
| **LysoPC a C24:0** | 0.443 | 0.328 | 0.389 | 0.955 | 0.460 | 0.413 |
| **LysoPC a C28:0** | 0.878 | 0.717 | 0.855 | 0.770 | 0.758 | 0.844 |
| **Phosphatidylcholines** | | | | | | |
| **PC aa C24:0** | 16.898 | 15.214 | 11.362 | 37.606 | 11.249 | 11.223 |
| **PC aa C26:0** | 2.284 | 4.219 | 2.879 | 4.568 | 1.953 | 1.500 |
| **PC ae C30:0** | 0.737 | 0.711 | 0.947 | 1.221 | 0.570 | 0.657 |
| **PC aa C30:2** | 2.099 | 2.435 | 2.955 | 6.189 | 1.569 | 1.293 |
| **PC ae C32:2** | 2.057 | 1.658 | 2.315 | 3.689 | 1.887 | 2.515 |
| **PC ae C32:1** | 0.989 | 0.829 | 1.159 | 1.613 | 0.859 | 0.911 |
| **PC aa C32:2** | 0.847 | 0.813 | 0.797 | 0.816 | 0.798 | 0.778 |
| **PC aa C32:1** | 0.635 | 0.422 | 0.458 | 0.641 | 0.425 | 0.621 |
| **PC aa C32:0** | 0.277 | 0.260 | 0.248 | 0.311 | 0.212 | 0.331 |
| **PC ae C34:3** | 0.638 | 0.342 | 0.222 | 0.704 | 0.347 | 0.484 |
| **PC ae C34:2** | 0.976 | 0.814 | 0.797 | 0.972 | 0.915 | 0.982 |
| **PC ae C34:1** | 0.729 | 0.415 | 0.612 | 1.097 | 0.497 | 0.610 |
| **PC ae C34:0** | 1.815 | 1.644 | 1.503 | 3.119 | 1.464 | 1.586 |
| **PC aa C34:4** | 1.366 | 1.075 | 0.910 | 2.257 | 1.083 | 1.470 |
| **PC aa C34:3** | 4.935 | 3.140 | 2.887 | 10.563 | 3.807 | 4.707 |
| **PC aa C34:2** | 13.458 | 10.643 | 7.267 | 29.422 | 6.589 | 4.835 |
| **PC aa C34:1** | 57.090 | 42.686 | 38.241 | 120.210 | 36.401 | 39.343 |
| **PC ae C36:5** | 12.946 | 8.383 | 9.252 | 34.454 | 6.613 | 5.320 |
| **PC ae C36:4** | 23.286 | 21.412 | 21.151 | 51.549 | 16.831 | 21.413 |
| **PC ae C36:3** | 14.483 | 16.708 | 17.633 | 36.297 | 12.960 | 18.492 |
| **PC ae C36:2** | 6.741 | 7.962 | 9.460 | 15.968 | 6.202 | 9.486 |
| **PC ae C36:1** | 1.150 | 1.025 | 1.122 | 2.693 | 0.976 | 1.215 |
| **PC aa C36:3** | 3.633 | 3.727 | 4.180 | 10.827 | 3.204 | 3.931 |
| **PC aa C36:2** | 7.349 | 7.828 | 9.170 | 17.452 | 7.310 | 12.473 |
| **PC aa C36:1** | 18.067 | 17.629 | 17.236 | 37.164 | 16.954 | 27.358 |
| **PC aa C36:0** | 24.732 | 19.604 | 19.410 | 55.465 | 21.910 | 32.429 |
| **PC ae C38:6** | 8.998 | 8.627 | 9.281 | 24.541 | 8.552 | 10.994 |
| **PC ae C38:0** | 1.363 | 1.112 | 1.184 | 2.095 | 1.053 | 1.000 |
| **PC aa C38:6** | 8.594 | 8.244 | 8.291 | 16.869 | 7.394 | 15.837 |
| **PC aa C38:3** | 23.767 | 16.708 | 19.410 | 49.096 | 19.613 | 25.603 |
| **PC aa C38:1** | 3.992 | 10.946 | 8.274 | 7.806 | 5.424 | 4.737 |
| **PC ae C40:1** | 2.138 | 2.056 | 3.527 | 2.983 | 2.360 | 3.230 |
| **PC aa C40:6** | 2.077 | 2.724 | 4.424 | 2.953 | 3.700 | 2.948 |
| **PC aa C40:3** | 0.357 | 6.879 | 20.034 | 0.802 | 1.129 | 0.712 |
| **PC aa C40:1** | 2.268 | 1.912 | 2.512 | 4.147 | 1.902 | 2.420 |
| **PC ae C42:5** | 0.357 | 0.598 | 1.453 | 0.715 | 0.375 | 0.398 |
| **PC ae C42:4** | 3.412 | 2.285 | 2.434 | 6.320 | 2.408 | 2.844 |
| **PC ae C42:3** | 2.836 | 2.021 | 2.544 | 5.246 | 2.729 | 3.385 |
| **PC ae C42:2** | 1.128 | 0.732 | 1.169 | 2.071 | 1.141 | 1.324 |
| **PC ae C42:1** | 0.344 | 0.354 | 0.519 | 0.513 | 0.366 | 0.454 |
| **PC aa C42:4** | 6.125 | 2.261 | 2.223 | 9.309 | 2.978 | 2.716 |
| **PC aa C42:2** | 2.051 | 1.163 | 0.984 | 4.348 | 2.035 | 2.114 |
| **PC aa C42:1** | 0.471 | 0.302 | 0.326 | 0.850 | 0.306 | 0.457 |

7, 14 and 21= days after inoculation.
